# Enhancing the Interpretation of Competition Performance and Physical Characteristics in Male Youth Track and Field Athletes

**DOI:** 10.1002/ejsc.70259

**Published:** 2026-09-30

**Authors:** James Baker, Paul Read, Philip Graham‐Smith, Mauricio Monaco, Marco Cardinale, Thomas W. Jones

**Affiliations:** ^1^ Aspire Academy for Sport Excellence Doha Qatar; ^2^ Department of Sport Exercise and Rehabilitation Northumbria University Newcastle Upon Tyne UK; ^3^ London Sports Institute Middlesex University Dubai UAE; ^4^ School of Sport and Exercise University of Gloucestershire Gloucester UK; ^5^ Division of Surgery and Interventional Science University College London London UK; ^6^ Department of Sport & Exercise Sciences Institute of Sport Manchester Metropolitan University Manchester UK; ^7^ Aspetar Qatar Orthopaedic and Sports Medicine Hospital Doha Qatar

**Keywords:** athletics, growth and maturation, talent identification

## Abstract

This retrospective study examined the associations of maturity status and chronological age with competition and physical testing outcomes in youth track and field athletes, and evaluated corrective adjustment procedures for competition outcomes. Data were collected over five seasons from 75 male athletes (13.9 ± 1.3 years; percentage predicted adult height (%PAH) 93.3 ± 4.6%; height 164.1 ± 10.1 cm; body mass 51.4 ± 10.4 kg) for 60 m sprint, long jump (LJ), countermovement jump, isometric leg press, 10–5 repeated jump test, and 50 m sprint outcomes. Linear mixed models (LMMs) quantified the individual associations of maturity status and chronological age with each outcome. Model‐derived fixed‐effect coefficients were used to apply mean‐ and sample‐maximum corrections to 60 m and LJ performances. Mean corrections were effective at reducing maturity‐performance relationships for the 60 m (*r* = −0.78 to −0.11, *R* ^2^ = 0.61 to 0.01) and the LJ (*r* = 0.63 to 0.01, *R* ^2^ = 0.40 to < 0.001) when the correction reflected the LMM findings. Sample maximum corrections demonstrated stronger agreement and lower error for the 60 m (*R* ^2^ = 0.80; RMSE = 0.19 s) than LJ (*R* ^2^ = 0.38; RMSE = 0.43 m). These findings support outcome‐specific mean corrections for enhancing the interpretation of male youth track and field performance, while sample‐maximum corrected performances should be treated as estimates and presented with an appropriate measure of prediction error.

## Highlights

- Linear mixed models incorporating both maturity status and chronological age showed outcome‐specific associations with competition and physical testing outcomes in male youth track and field athletes.
- Corrective adjustment procedures should be outcome‐specific and reflect the relative contribution of maturity status and chronological age identified within each model.
- Mean corrections substantially reduced maturity‐performance relationships in the 60 m and long jump, when the correction reflected the corresponding LMM findings.
- Sample‐maximum corrections showed stronger agreement and lower error for 60 m than long jump; corrected performances should therefore be presented with an appropriate measure of error.

## 1 Introduction

Track and field success is largely dependent on high levels of speed, power and strength, particularly in sprinting, throwing and jumping events (Graham‐Smith and Brice 2010; Weyand et al. 2000; Rabita et al. 2015). During adolescence, these physical qualities are influenced by chronological age and biological maturation (Meyers et al. 2015; Croix et al. 1999; Radnor et al. 2017, 2022; Lloyd et al. 2011; Baker et al. 2025, 2021). Chronological age reflects time‐related development and progression through age‐graded training and competition environments. In academy settings, it may also partly capture accumulated exposure to structured training, coaching, physical preparation, testing, and competition. In contrast, maturity status reflects an athlete's current stage of biological maturation (Malina et al. 2004). Large inter‐individual differences can exist in maturity status, rate and timing of growth and maturation, especially between 12 and 15 years of age for boys (Malina et al. 2015). These maturity‐related differences have been shown to impact youth track and field performance (Baker et al. 2024). Consequently, less mature athletes may be disadvantaged during age‐group competition or physical testing. This may increase the risk of them being overlooked or deselected from talent development programs or scholarships if maturity‐related differences and future performance potential are not considered.

Competitive opportunities are available at very early stages of an athlete's development in track and field. Athletes can compete within chronological age groups up to the Under 23 level. National competitions typically commence from the age of 14 and international events between 14 and 16 years. However, evidence suggests that early youth success is a poor predictor of senior success, with only a small proportion of highly ranked youth athletes maintaining comparable rankings at a senior level (Kearney and Hayes 2018). Within age‐groups, age‐related differences have been reported to influence performance outcomes in track and field (Bezuglov et al. 2022; Brustio et al. 2019; Difernand et al. 2023). Interpreting performance differences at a youth level is further complicated by biological maturation, with evidence from other youth sports showing differences up to 6 years in skeletal age between the least and most mature athletes within the same chronological age group (Whiteley et al. 2017). Competition results are typically interpreted with reference to age‐group benchmarks or qualifying standards. These developmental differences may contribute to biased selection or deselection decisions (Till et al. 2018) and may partly explain why progression rates from youth to senior level are relatively low (Kearney and Hayes 2018; Boccia et al. 2021a, 2021b).

A recent meta‐analysis reported moderate to large differences in sprint and jump performance between youth athletes grouped as pre‐, circa‐ and post‐Peak Height Velocity (PHV) (Baker et al. 2025). Similarly, in male youth track and field athletes, significant increases in 60 m and long jump performance with large to very large effect sizes have been observed between the least mature group (approaching‐PHV, 85%–90% predicted adult height [%PAH]) and the most mature group (post‐PHV, > 95 %PAH) (Baker et al. 2024). The post‐PHV group also significantly outperformed the approaching‐ and circa‐PHV groups on almost all speed, power, and strength measures (Baker et al. 2024). These differences between maturity groups are largely related to age‐, growth‐, and maturity‐related changes in testosterone levels (Handelsman et al. 2018), muscle size (Croix et al. 2002; Lexell et al. 1992; Kannas et al. 2010), fibre type (Gollnick and Matoba 1984; Oertel 1988), muscle architecture (Radnor et al. 2022; Kannas et al. 2010; Binzoni et al. 2004), muscle activation (Falk et al. 2009; Grosset et al. 2008), mechanical tendon properties (Kubo, Teshima, Hirose, et al. 2014; Kubo, Teshima, Ikebukuro, et al. 2014, 2001), and neuromuscular capacity (Lloyd et al. 2012). Anthropometric (i.e., leg length) changes also contribute to enhanced speed by increasing stride length in more mature athletes (Meyers et al. 2017). Therefore, accurately evaluating performance in youth age groups requires consideration of differences in maturity status alongside chronological age.

Although these findings demonstrate the influence of maturity status on competition performance and physical test outcomes, the use of maturity group comparisons has limitations. Grouping athletes by %PAH thresholds does not account for chronological age, despite age differences between maturity groups and potentially contributing to performance differences. In addition, some outcomes did not differ significantly between the least mature groups (Baker et al. 2024), suggesting that maturity‐group comparisons may not capture the combined influence of age‐ and maturity‐related development. Categorizing a continuous variable, such as %PAH, can also reduce statistical power and contribute to residual confounding (Royston et al. 2006). Furthermore, maturity estimates derived from somatic measures, such as %PAH and maturity offset (years from PHV), are subject to prediction error, which may influence maturity classification and any subsequent analyses or application that uses them (Mirwald et al. 2002; Lolli et al. 2022, 2021). Therefore, modelling chronological age and maturity status as continuous predictors may provide a more appropriate approach to examine their independent associations with competition performance and physical testing outcomes.

Various strategies have been developed to account for inter‐individual age and maturity differences when evaluating competition performance and physical characteristics. Sixty metre sprint and long jump maturity group benchmarks have recently been published for young male athletes (Baker et al. 2024). Model‐based approaches provide an alternative to categorical group comparisons by retaining age and maturity status as continuous variables, avoiding the negative statistical impact of converting these variables to categorical groups (Royston et al. 2006). Corrective adjustment procedures use regression‐derived estimates to adjust performance to a common age or maturity reference point, thereby reducing age‐ or maturity‐related bias when comparing youth athletes (Cobley et al. 2019; Hogan et al. 2022; Charbonnet et al. 2022; Romann and Cobley 2015). Previous research has applied the procedures to account for relative age differences in youth swimming (Cobley et al. 2019) and sprinting (Romann and Cobley 2015), and maturity‐related differences using maturity offset in youth swimming (Hogan et al. 2022) and soccer (Charbonnet et al. 2022). Two variations of maturity corrective adjustment procedures have previously been investigated. Mean corrections which anchor corrected performances to the average maturity status of the sample and allow athletes to be compared at a common maturity reference point (Charbonnet et al. 2022). Sample‐maximum corrections, adjust performances to a more advanced maturity status, typically the highest maturity status within a sample of athletes and may therefore provide an estimate of performance at a more advanced maturity status (Abbott et al. 2021). Model‐based Z‐scores have also been used in previous research, to compare an athlete's observed performance with their expected performance for their chronological age or maturity status (Till et al. 2018).

However, there is limited evidence of corrective adjustment procedures being applied in youth track and field athletes. Although age‐based corrections have been applied in sprinting (Romann and Cobley 2015), mean‐ and sample‐maximum maturity‐based corrections have not yet been applied to youth track and field competition outcomes. Therefore, it remains unclear whether mean corrections reduce maturity‐ and/or age‐related differences in this population or how accurately sample‐maximum corrections estimate subsequent competition performance. This represents an important gap because failing to account for these developmental differences may compromise the interpretation of current performance when competition results are used within talent identification and development processes.

Accordingly, the present study addressed the following three research questions:

RQ1: What are the independent associations of maturity status, estimated using skeletal‐age‐derived %PAH, and chronological age with competition and physical test outcomes?

RQ2: Do event‐specific mean corrections reduce maturity‐performance relationships in 60 m and long jump?

RQ3: How well do sample‐maximum corrections estimate subsequent 60 m and LJ performance in terms of agreement and prediction error?

In addition to addressing these research questions, we present an applied example with the sample‐maximum corrections to estimate performance at a more advanced maturity status with model‐based *Z* scores to contextualize performance relative to that expected for the athlete's maturity status and chronological age.

## 2 Materials and Methods

### 2.1 Study Design

A retrospective observational study design was used to examine routinely collected academy competition and physical testing data over five seasons (2017–2022). Data were included from athletes enrolled in the academy during their first 3 years (grade 7 to 9, age 12–16). The dataset included a mixture of single and repeated observations per athlete, reflecting the academy context where athlete availability varied due to injury, selection or deselection, and progression through age groups. Repeated observations were retained where possible and accounted for in the statistical models. Inclusion required the competition results (60 m and LJ) and physical tests (50 m sprint, CMJ, 10‐5 RJT and ILP) to have been collected within 31 days of the maturity assessment to reduce the likelihood of changes in the maturity status of the athlete between the assessment of maturation and time of testing and competition.

### 2.2 Subjects

Seventy‐five trained, male youths (age 13.9 ± 1.3 years; 93.3 ± 4.6 %PAH; stature 164.1 ± 10.1 cm; body mass 51.4 ± 10.4 kg) enrolled in an elite development academy for track and field (athletics) with exposure of up to 16 hours a week of structured training that incorporated technical training, strength and conditioning and multi‐sport sessions. Ethical approval was obtained (E20140000012) from Aspire Zone Foundation Institutional Review Board and Research Committee as part of a larger study on growth and maturation in youth athletes. Parental consent and participant assent were obtained prior to commencement.

Mean corrections were applied to the full available competition datasets for 60 m and LJ. The 60 m dataset included 99 observations from 63 athletes (age 13.1 ± 0.8 years; 91.1 ± 4.3 %PAH; height 159.9 ± 9.4 cm; body mass 46.6 ± 8.6 kg), while the LJ dataset included 138 observations from 63 athletes (age 13.1 ± 0.9 years; 91.1 ± 4.6 %PAH; height 159.9 ± 8.7 cm; body mass 46.8 ± 9.0 kg). Descriptive statistics were calculated from each athlete's first observation.

Twenty‐eight athletes with a minimum of 2 repeated observations available for the 60 m and/or LJ competition results were used to assess the agreement and prediction error of the sample‐maximum maturity corrective adjustment procedures, providing 84 total observations [60 *m* = 38; LJ = 46]. Descriptive statistics for the group were calculated from their first observation (*n* = 28; age 12.9 ± 0.7 years; 91.2 ± 4.1 %PAH; height 160.7 ± 9.4 cm; body mass 47.7 ± 8.5 kg). After this assessment was completed, the sample‐maximum correction was applied to results from an under‐14 60 m competition (*n* = 9; age 13.3 ± 0.3 years; 93.2 ± 6.7 %PAH; height 162.5 ± 5.7 cm; body mass 49.1 ± 6.7 kg) and long jump competition (*n* = 9; age 13.6 ± 0.2 years; 95.0 ± 3.1 %PAH; height 167.8 ± 5.9 cm; body mass 54.2 ± 4.7 kg). The U14 age group was selected because it coincides with the expected period of peak height velocity (PHV) in boys (Rogol et al. 2002; Tanner et al. 1966).

### 2.3 Procedures

#### 2.3.1 Anthropometry

Stature, sitting height, and body mass were recorded on a calibrated measurement platform (Harpenden Stadiometer, Holtain Limited, Crosswell, UK) and weighing scale (M304601, ADE Hamburg, Germany), respectively.

#### 2.3.2 Adult Predicted Height

Skeletal age, and subsequently, the calculation of predicted adult height was determined using hand‐wrist radiographs, standing height, and body mass measurements. Skeletal age was assessed using standard radiographs (Digital Diagnost; Philips, USA) (Malina 2011). Roentgenograms were evaluated by automated processing of digital images using the computerized BoneXpert software (version 3.1.4, Visiana, Holte, Denmark) (Thodberg et al. 2012) to provide the adult predicted height. The specific details of these methods have been published previously (Lolli et al. 2021).

#### 2.3.3 Maturity Status

Maturation status was determined by calculating each participant's current percentage of predicted adult height %PAH. This was achieved using the final adult predicted height as provided by the BoneXpert software with the stature measurements obtained at the time of testing and competition. The following formula was applied:

$$\frac{Current\;stature\;\left(cm\right)}{Bone\;Xpert\;Predicted\;Adult\;Height\;\left(cm\right)}\;x\;100=\%\;Predicted\;Adult\;Height$$

#### 2.3.4 Competition Performance (60 m and Long Jump)

Results were collected during official indoor athletic competitions for which the lead investigator was present at all the events. Indoor competition was chosen because it provided controlled conditions without environmental influence (e.g., wind, temperature and humidity). The sixty‐metre sprint (60 m) and long jump (LJ) events took place on an indoor athletics track that met the IAAF standards for international competition. The 60 m event was electronically timed using the FinishLynx photo‐finish technology timing system (Haverhill, MA, USA). Further details of the methods applied to the competition data have been published previously (Baker et al. 2024).

In addition to the competition outcomes, routinely collected physical testing outcomes were included to examine the associations of chronological age and maturity status with physical performance measures that have previously been identified as variables of importance for projection in 60 m and/or LJ performance in a male youth track and field cohort (Baker et al. 2024). Corrective adjustment procedures were evaluated for the competition outcomes only; the physical testing outcomes are presented to support potential future applications of the model‐derived coefficients.

#### 2.3.5 Countermovement Jump

Participants performed three maximal countermovement jumps with their hands placed on their hips on a dual‐force platform system (ForceDecks FD4000, Vald Performance, Brisbane, Australia) sampling at 1000 Hz (Bosco et al. 1981). They were instructed to stand still prior to being given the command to ‘jump as high as possible’. All 3 jumps were completed within 60s separated by ∼ 20s of rest between each. CMJ jump height (CMJ JH) and relative power (RP) were extracted for analysis.

#### 2.3.6 50 m Sprint

Three maximal 50 m sprints were performed whilst being tracked using a laser speed gun sampling at 100 Hz (Laveg LDM 300C, Jenoptik, Jena), positioned approximately 3m behind the zero line. Participants performed the sprints individually, starting each run in an upright position 0.5 m behind the zero line. The variables extracted for analysis included: (0–50 m), (0–40 m), (0–30 m), (0–20 m), (0–10 m), (0–5 m) and maximum instantaneous velocity.

#### 2.3.7 10‐5 Repeated Jump Test

The 10‐5 RJT was completed using a contact jump mat (Smartspeed, Vald Performance, Brisbane, Australia). The test was initiated with one countermovement jump, followed by 10 consecutive reactive maximal jumps with hands on the hips throughout. Participants were instructed to jump ‘as high as possible and minimize the time spent on the ground’. The five jump with the highest flight time (FT) and ground contact time (GCT) < 250 ms were used for the analysis. The reactive strength index (RSI) was calculated as the FT divided by the GCT (both in milliseconds). The mean RSI, FT, GCT, and jump height (JH) were calculated for the five selected jumps. Athletes completed two trials with 3 minutes rest. The trial with the highest mean RSI was used in the analysis. Further details of the methods, validity, and reliability of the 10‐5 RJT protocol have been previously published (Baker et al. 2021).

#### 2.3.8 Isometric Leg Press

A 45‐degree leg press (Plated Loaded Linear Leg Press, Hammer Strength, Des Plaines, IL, USA) was adapted with a 60 × 40 cm single force plate (Kistler, model 9281E, Winterthur, Switzerland) to assess the isometric leg extension peak. The knee angle was set to 110° using a manual goniometer. Athletes worked through a progressive warm up at a self‐determined 50%, 70% and 90% of maximum effort for 5 s, with at least 1 min rest between each. Two maximal trials were completed, with at least 3‐min between trials. The trial with the highest peak force was used for analysis. Full details of the methods applied in the ILP assessment have been published previously (Baker et al. 2024).

### 2.4 Statistical Analysis

An exploratory data analysis (EDA) was undertaken to ensure data integrity and assess model suitability. Missing data and duplicate athlete‐session entries were identified and removed. Outliers were identified using box plots and reviewed to determine if they were measurement errors or true extreme performances. Linearity between maturity status (%PAH) was assessed by visual inspection of scatter plots with linear and locally estimated smoothing (LOESS). Residual distributions and variance patterns were visually assessed using histograms, Q‐Q plots, and residual versus fitted plots to identify any deviation from normality or unequal variance that might affect model performance. Sample size, total observations, mean observations per athlete, and range were reported for each outcome, confirming repeated measures within the dataset (see Table 1). Linear mixed models (LMMs) were used to investigate the associations between maturity status and chronological age and the outcome variables. LMMs were chosen because the data were hierarchically structured, and it is possible to account for multiple observations of athletes within the sample. Intraclass correlation coefficients (ICCs) were calculated to quantify clustering effects, with values > 0.40 indicating substantial between‐athlete variance. In the present study, ICCs ranged from 0.45 to 0.81 across outcomes, confirming the need for random effects to accurately model variability and avoid biased standard errors.

**TABLE 1:** Linear mixed‐model outputs for competition and physical testing outcomes including fixed effects, model fit and error estimates

Outcome	N	Obs.	Mean obs.	Intercept	%PAH slope	95% CI (%PAH)	Model mean %PAH	*p* (%PAH)	Age slope	95% CI (Age)	Model mean Age (years)	*p* (Age)	Residual SD	SEE (fixed)	SEE (conditional)	ICC	AIC	BIC	VIF
60 m time (s)	63	99	1.57	8.60	−0.105	[−0.14, −0.08]	92.68	0.000	−0.06	[−0.18, 0.07]	13.47	0.355	0.19	0.43	0.13	0.81	96.0	109.0	4.18
Long jump (m)	63	138	2.19	4.38	0.047	[0.02, 0.08]	92.36	0.002	0.26	[0.13, 0.38]	13.36	< 0.001	0.30	0.43	0.25	0.53	153.1	167.7	2.53
CMJ height (m)	71	191	2.69	0.33	0.003	[0.001, 0.006]	92.81	0.003	0.03	[0.02, 0.04]	13.66	< 0.001	0.03	0.04	0.02	0.60	−674.8	−658.6	2.93
CMJ relative power (W·kg^−1^)	71	191	2.69	50.56	0.51	[0.21, 0.81]	92.81	0.001	2.79	[1.50, 4.08]	13.66	< 0.001	3.28	5.28	2.74	0.67	1122.8	1139.1	3.12
Max velocity (m·s^−1^)	38	58	1.53	8.55	0.055	[0.002, 0.11]	94.86	0.043	0.34	[0.16, 0.52]	14.21	< 0.001	0.23	0.49	0.15	0.80	84.5	94.8	2.09
10 m time (s)	38	58	1.53	1.78	−0.03	[−0.06, −0.01]	94.86	0.010	−0.03	[−0.06, 0.01]	14.21	0.003	0.04	0.06	0.03	0.54	−131.0	−120.7	1.79
20 m time (s)	38	58	1.53	3.10	−0.07	[−0.11, −0.02]	94.86	0.005	−0.06	[−0.11, −0.02]	14.21	0.003	0.08	0.11	0.06	0.53	−63.2	−52.9	1.78
30 m time (s)	38	58	1.53	4.35	−0.02	[−0.05, −0.009]	94.86	0.004	−0.10	[−0.18, −0.05]	14.21	0.002	0.12	0.17	0.09	0.59	−19.1	−8.8	1.83
40 m time (s)	38	58	1.53	5.59	−0.041	[−0.01, −0.07]	94.86	0.004	−0.17	[−0.26, −0.07]	14.21	0.001	0.15	0.24	0.11	0.65	16.8	27.1	1.88
50 m time (s)	38	58	1.53	6.86	−0.058	[−0.09, −0.02]	94.86	0.003	−0.21	[−0.34, −0.07]	14.21	0.003	0.22	0.33	0.16	0.61	52.7	63.0	1.84
ILP peak force (N)	56	111	1.98	2498.46	182.32	[−26.37, 391.0]	93.32	0.086	448.7	[245.21, 652.19]	13.63	< 0.001	449.33	729.15	352.82	0.63	1724.0	1737.5	2.76
10–5 RJT jump height (cm)	75	202	2.69	29.37	0.56	[−0.53, 1.65]	93.28	0.310	3.72	[2.61, 4.83]	13.88	< 0.001	3.33	4.38	2.89	0.46	1148.7	1165.3	2.52
10–5 RJT RSI (FT/CT)	75	202	2.69	2.89	−0.02	[−0.13, 0.08]	93.28	0.690	0.22	[0.10, 0.33]	13.88	< 0.001	0.34	0.46	0.30	0.45	240.9	257.4	2.51
10–5 RJT flight time (ms)	75	202	2.69	486.77	1.37	[−0.65, 3.38]	93.28	0.180	23.21	[15.91, 30.51]	13.88	< 0.001	28.36	36.93	24.68	0.45	1999.3	2015.9	2.51

Abbreviations: %PAH = percentage predicted adult height; AIC = Akaike information criterion; BIC = Bayesian information criterion; CMJ = countermovement jump; CT = contact time; FT = flight time; ICC = intraclass correlation coefficient; ILP = isometric leg press; Obs. = observations; RJT – repeated jump test; RSI = reactive strength index; SEE = standard error of the estimate; VIF = variance inflation factors.

The following model fitting, residual diagnostics were performed to confirm that the assumptions were met. The normality of residuals was evaluated using Q–Q plots and the Shapiro–Wilk test. Homoscedasticity was visually inspected via residual versus fitted and scale‐location plots. Influential observations were identified by ranking absolute residuals and were reviewed to confirm data integrity.

Each LMM included fixed effects for maturity status (%PAH) and chronological age (decimal years), both modelled continuously, with random intercepts for individual athletes to account for within athlete correlation. Collinearity between chronological age and maturity status (%PAH) was assessed using variance inflation factors (VIF) for all outcome variables (Table 1). VIF values ranged from 1.78 to 4.18, which is acceptable in line published collinearity thresholds (VIF < 5) (Hair et al. 2019) supporting the inclusion of both predictors in the same model.

Two model structures were considered for each outcome: random intercept and random slope models. Random intercept models account for baseline differences between athletes, while random slope models allow the relationship between maturity status (%PAH) and performance to vary across individuals. Random slope models failed to converge across all outcomes, likely due to insufficient repeated measures per athlete (mean observations per variable ranged from 1.52 to 2.69; full data are presented in Table 1). Therefore, random intercept models were used for the final analyses because they provided a stable and interpretable approach for accounting for repeated observations with athletes. Akaike Information Criterion (AIC) and Bayesian Information Criterion (BIC) for the models are presented in Table 1.

For each outcome, the model estimated an intercept (representing baseline performance) and fixed effect slopes for maturity status and chronological age. Maturity status and age were mean‐centred, so the model's intercept represents the expected performance at the mean of the sample, improving the interpretability of coefficients and adjusted values. Centring also reduces multicollinearity, stabilises model estimation and convergence, and improves the precision of fixed‐effect slope estimates from the LMMs. Model fit was evaluated using conditional *R* ^2^ (*R* ^2^c) to quantify the variance explained by the fixed and random effects combined, and marginal *R* ^2^ represents the variance explained by the fixed effects alone (Nakagawa and Schielzeth 2013). The contribution of these fixed effects was evaluated further using regression coefficients, *p* values (< 0.05) and partial *R* ^2^ to determine unique variance explained by each predictor after accounting for the other fixed‐effects in the model. Model error was quantified using the standard error of the estimate (SEE), with SEE fixed representing the potential error based on only fixed effects and SEE conditional representing error when fixed and random effects are included, accounting for individual athlete variability across repeated measures. 10‐5 RJT contact time was excluded from the analysis because the test protocol applied a constraint of < 250 ms for inclusion. Exploratory data analyses and linear mixed models were performed using R (version 4.5.1, released 2025‐06‐13; R Foundation for Statistical Computing, Vienna, Austria) and RStudio (version 2025.05.1 + 513; Posit Software, Boston, MA, USA) with the lme4, lmerTest and performance packages.

The effectiveness of maturity corrective adjustment procedures (Mat‐CAPs) has previously been established (Charbonnet et al. 2022; Romann and Cobley 2015; Abbott et al. 2021) and a four‐step process to create the corrected scores was outlined by Charbonnet and colleagues (Charbonnet et al. 2022). Due to the repeated observations in the present study, this method was adapted to use regression coefficients derived from the LMMs rather than linear models in the original study. The LMMs provided intercept, maturity status (%PAH) slope and age slope estimates that were used to generate corrected 60 m times and long jump results. These regression coefficients were used to adjust each athlete's actual performance using the following steps:

1. Calculate an expected score at the anchor (ESA) at the model mean or sample‐maximum:

$$ESA=b_{0}^{\left(c\right)}+\beta_{PAH}\;\left(A_{PAH}-\mu_{m}\right)+\beta_{age}\;\left(A_{age}-\mu_{a}\right)$$

2 Calculate an expected raw score (ERS) for the athlete's observed maturity status and age:

$$ERS=b_{0}^{\left(c\right)}+\beta_{PAH}\;\left(\%PAH-\mu_{m}\right)+\beta_{age}\;\left(Age-\mu_{a}\right)$$

3 Calculate the event‐specific correction factor (CF) and corrected score (CS):
  a 60 m (time; multiplicative method):
$$CF=\frac{RS}{ERS},\;CS=ESA\times CF$$
  b. LJ (distance; additive method)
r=RS−ERS, CS=ESA+r

For 60 m, a multiplicative approach for the CS was used to preserve proportional (percent) differences; for LJ, the additive correction was selected to create the CS by adding the residual to preserve absolute difference in metres. The additive method was empirically supported because when agreement and prediction error were assessed, the multiplicative approach showed significant negative bias and larger errors (mean bias −0.92 ± 0.42, limits of agreement [LOA] −0.09 to −1.75, mean absolute error [MAE] 0.92 m, root mean square error [RMSE] 1.01 m).

### 2.5 Formula Notations

CS: corrected score at the chosen anchor

RS: raw score (s for 60 m; *m* for LJ)

*b*_0_^(c)^: centred intercept

ß_PAH,_ ß_age_: maturity (per 1% PAH) and age (per 1 year) slopes

μ_*m*_, μ_*a*_: maturity and age model means used for centring

A_PAH_: maturity anchor (e.g., model mean or sample maximum)

A_age_: age anchor (e.g., model mean or known value at subsequent observation)

The corrections were applied in two ways. First, the mean correction (Charbonnet et al. 2022) was used to correct for maturity‐ and, where appropriate, age‐related differences in the 60 m and LJ outcomes. The variables included in each correction were determined by the LMM outputs. Where maturity status was the only significant fixed‐effect, the model mean %PAH was used as the correction anchor. Where both maturity status and chronological age were significant fixed effects, the model mean %PAH and model mean age were used together. The effectiveness of the correction was assessed by the change in the correlation between %PAH and performance before and after the correction.

Second, the sample‐maximum correction was examined, in which a performance is corrected to a more advanced maturity status. To assess the agreement and error of the sample‐maximum corrections, repeated observations were used where available. Earlier performances were corrected to the athlete's %PAH at a subsequent time point where an actual competition result was available, with chronological age held at the model mean. The predicted value was then compared with the corresponding actual result. Association was quantified with Pearson's *r*; agreement was evaluated using Bland–Altman (bias = Actual–Predicted), MAE and RMSE. Calibration was assessed by regressing Actual on Predicted (*Actual = a + b·Predicted*); when the intercept differed from 0 and/or the slope from 1, a post hoc linear recalibration was applied (*Adjusted = a + b·Predicted*), after which agreement metrics were recalculated.

Following the assessment of agreement and error, the sample‐maximum correction was applied to a sample of U14 athletes for the 60 m and LJ, using the highest %PAH within a 12‐month age group sample as the maturity anchor (Abbott et al. 2021). RMSE was presented alongside these corrections to show the potential error in the estimates. For this approach, anchor age was held at the model mean, given that it is impossible to identify the specific age at which a certain %PAH will occur. In addition, to present another practical example of the utility of the modelling approach, model‐based Z‐scores were used to quantify how a sample of raw performance compared with the expected value at the athlete's current maturity and age. Providing a benchmark of the level of performance against athletes of the same maturity status and chronological age. The following formula was utilized:

$$Model\;based\;Z\;Score=\frac{\left(RS-ERS\right)}{Residual\;SD}$$

All maturity corrective adjustment procedures, agreement and error analyses and model‐based Z‐score calculations were performed in Microsoft Excel (v16.76; Microsoft 365, Microsoft Corp., Redmond, WA, USA).

## 3 Results

Maturity status and chronological age demonstrated outcome‐specific associations with competition and physical testing performance. In competition outcomes, maturity status was associated with 60 m performance, whereas both chronological age and maturity status were associated with LJ performance. Their relative associations differed across physical testing outcomes. Visual inspection indicated that the relationships between maturity status (%PAH) and the outcome variables were linear. Residual plots and Q‐Q plots indicated no violations of normality or homoscedasticity, and Shapiro–Wilk tests supported the assumption of normally distributed residuals across all outcomes. ICCs ranged from 0.45 to 0.81, indicating moderate to substantial clustering of repeated measures within athletes. Full model statistics are provided in Tables 1 and 2.

**TABLE 2:** Conditional, marginal and partial R^2^ values from linear mixed models for linear mixed models of competition and physical testing outcomes

Outcome	*R* ^2^c	*R* ^2^m	Partial *R* ^2^: Maturity Status (%PAH)	Partial *R* ^2^: Chronological Age
60 m (s)	0.92	0.59	0.30	<0.01
Long jump (m)	0.74	0.45	0.10	0.11
CMJ jump height (m)	0.81	0.52	0.07	0.22
CMJ relative power (W·kg^−1^)	0.80	0.41	0.08	0.11
Max velocity (m·s^−1^)	0.89	0.43	0.09	0.19
10 m time (s)	0.76	0.48	0.16	0.17
20 m time (s)	0.76	0.49	0.18	0.17
30 m time (s)	0.80	0.50	0.18	0.18
40 m time (s)	0.83	0.52	0.18	0.19
50 m time (s)	0.80	0.50	0.19	0.16
ILP PF (N)	0.77	0.39	0.03	0.17
10–5 RJT jump height (cm)	0.71	0.45	< 0.01	0.24
10–5 RJT RSI (FT/CT)	0.54	0.16	< 0.01	0.09
10–5 RJT flight time (ms)	0.70	0.45	0.01	0.22

*Note:* Values are rounded to two decimal places; values < 0.01 are shown where appropriate.

Abbreviations: %PAH = percentage of predicted adult height attained; ILP = Isometric Leg Press PF = peak force; *R* ^2^c = conditional R^2^; *R* ^2^m = marginal R^2^; RJT = repeated jump test; RSI = reactive strength index;.

The LMM for the 60 m sprint explained 92% of the total variance in performance (*R* ^2^c = 0.92), with the fixed effects alone accounting for 59% (*R* ^2^m = 0.59). Faster 60 m sprint times were associated with higher maturity status as indicated by the significance of the %PAH slope (*ß* = −0.105, 95% CI [−0.14, −0.08]; *p* = < 0.001). Maturity status also made a substantial unique contribution to the model (partial *R* ^2^ = 0.30), whereas chronological age contributed negligibly and was not significant (*p* = 0.36). Accordingly, the mean correction for 60 m was applied using maturity status only.

The LMM for LJ explained 74% of the total variance (*R* ^2^c = 0.74), with the fixed effects alone accounting for 45% (*R* ^2^m = 0.45). Greater maturity status and chronological age were both associated with better LJ performance, as indicated by significant positive slopes of % PAH (*ß* = 0.047, 95% CI 0.02, 0.08; *p* = 0.002) and chronological age (*ß* = 0.26, 95% CI 0.13, 0.38; *p* = < 0.001). Partial *R* ^2^ values indicated similar unique contributions from maturity status (0.10) and chronological age (0.11), suggesting both contributed meaningfully to performance. Accordingly, the mean correction for the long jump was applied using both maturity status and chronological age. Full model statistics for both outcome variables are provided in Tables 1 and 2.

The LMMs explained between 76% and 89% of the variance in performance in the CMJ and 50 m split times and maximum velocity when fixed and random effects were combined (*R* ^2^c = 0.76–0.89), with fixed effects alone explaining 41%–52% of the variance (*R* ^2^m = 0.41–0.52). Both maturity status and age fixed‐effects were significant contributors across variables (all *p* < 0.05); higher maturity statuses and chronological ages were associated with increased jump heights and relative power in the CMJ and faster split times (10–50 m), and higher maximal velocity. Partial *R* ^2^ values indicated that maturity status and chronological age made similar contributions across the 50 m split times, whereas chronological age contributed more strongly than maturity status to CMJ performance and maximal velocity.

For the ILP and 10‐5 RJT, LMMs explained 54%–77% of the variance (*R* ^2^c = 0.54–0.77), while the fixed effects accounted for 16%–45% (*R* ^2^m = 0.16–0.45). In these outcomes, chronological age effect was the only consistent significant predictor (*p* < 0.001), with older athletes demonstrating greater ILP peak force, and higher jump height, flight time and RSI in the 10‐5 RJT. Partial *R* ^2^ values indicated that chronological age contributed substantially a more unique variance than maturity status across these measures. Model statistics for the physical tests are available in Tables 1 and 2.

SEE conditional was consistently lower than SEE fixed across all outcome variables, indicating that including random effects improved model precision (Table 1). For example, SEE decreased from 0.43 to 0.13 s in the 60‐m sprint time and from 0.43 to 0.25 m in the long jump. For CMJ JH and RP, the SEE decreased from 0.04 to 0.02 m, and 5.28 W·kg^−1^ to 2.74 W·kg^−1^, respectively.

Mean corrections were applied according to the LMM outputs for each competition outcome. The 60 m correction included maturity status only, whereas both maturity status and chronological age were included in the LJ correction. The effectiveness of the mean corrections in reducing the association between competition performance and maturity status is shown in Figures 1A,B and 2A,B for 60 m and LJ, respectively. Figure 1A shows that %PAH had a very strong negative correlation with 60 m time (*r* = −0.78, *R* ^2^ = 0.61) prior to correction. After applying the mean correction with maturity only (Figure 1B), this relationship was reduced to near‐trivial (*r* = −0.11) and the variance in 60 m time explained by maturity status fell from 61% pre‐correction (*R* ^2^ = 0.61) to 1% (*R* ^2^ = 0.01) post‐correction. Long jump showed a strong positive correlation with %PAH (*r* = 0.63, *R* ^2^ = 0.40) prior to correction (Figure 2A). After correction with both maturity status and age, it was effectively eliminated (*r* = 0.01, *R* ^2^ < 0.001) in Figure 2B.

**FIGURE 1:**
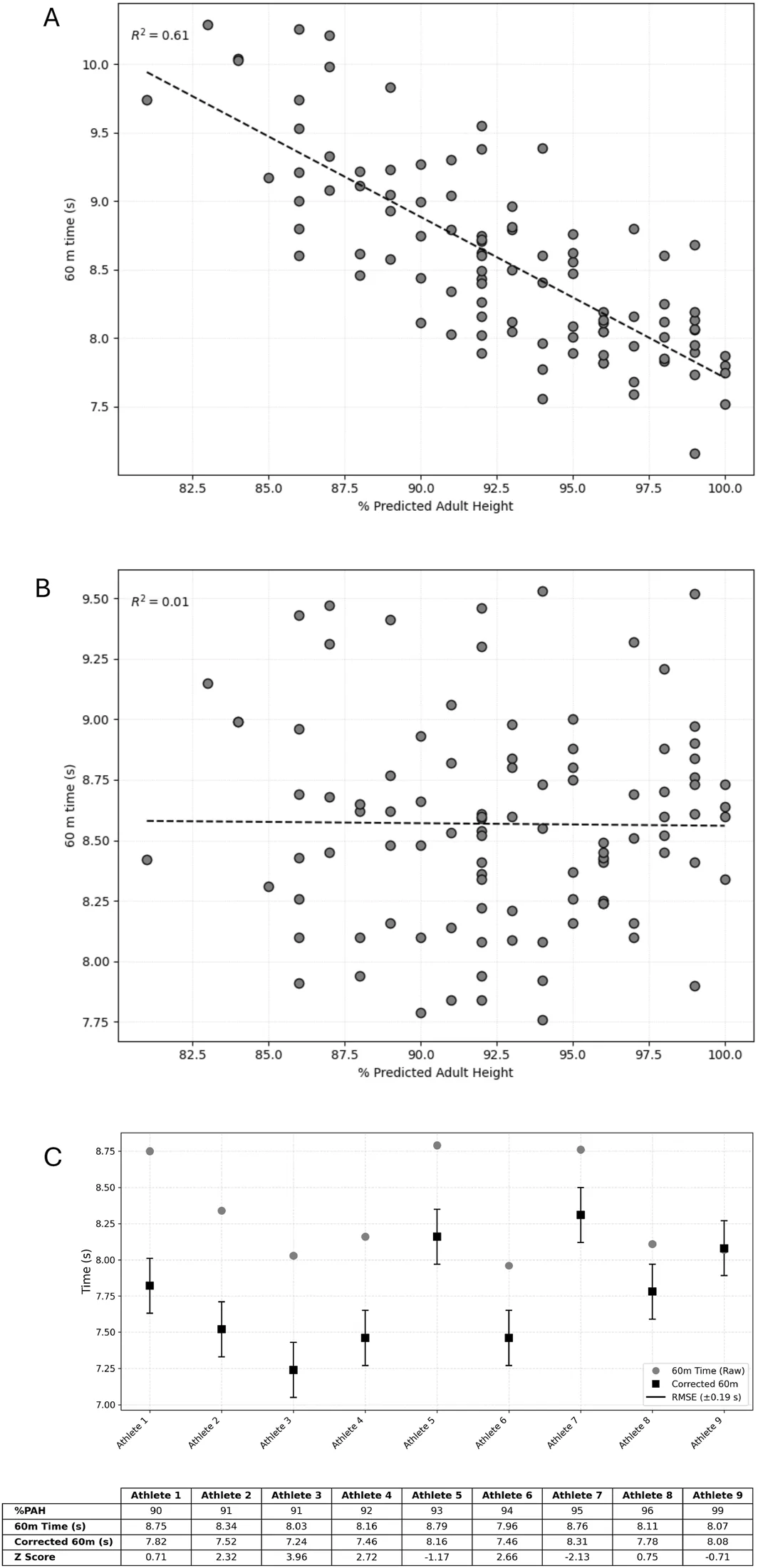
Relationships between maturity status and 60‐m sprint performance, and illustration of the maturity correction procedures. Panel (A): Correlation between %PAH and 60‐m time prior to correction. Panel (B): correlation between maturity status (%PAH), and 60‐m time post‐correction to the model mean maturity status only. Panel (C): presents 60‐m times for nine U14 athletes corrected to the sample maximum %PAH (99%). Grey circles represent raw times and black squares represent corrected times; vertical error bars indicate the RMSE (+0.19 s). The table reports each athlete's %PAH, raw and corrected times, and model‐based *Z* score for the uncorrected 60 m time.

**FIGURE 2:**
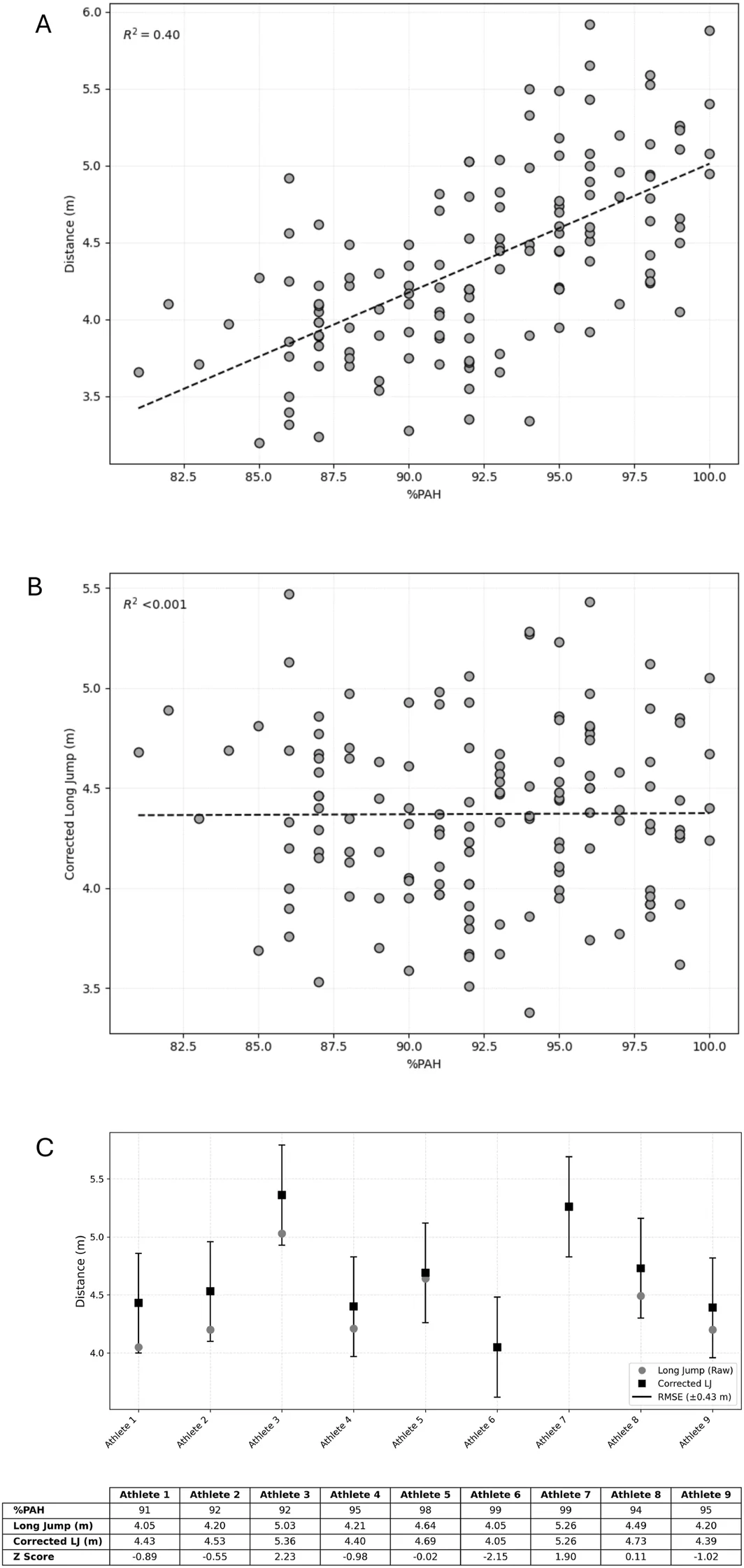
Relationships between maturity status and long jump performance, and illustration of the maturity correction procedures. Panel (A): correlation between maturity status (%PAH) and long jump distance prior to correction. Panel (B): correlation between %PAH and corrected long jump distance after correction to model mean maturity status and age. Panel (C): presents long jump distances for nine U14 athletes corrected to the sample maximum maturity status (99%PAH). Grey circles represent raw distances, and black squares represent corrected distances; vertical error bars indicate the RMSE (+0.43 m). The table reports each athlete's %PAH, raw and corrected distances, and model‐based *Z* score for the uncorrected long jump distance.

The assessment of agreement and prediction error for the sample‐maximum correction in the 60 m was based on 38 observations. Predicted and observed performance showed a strong association (*R* ^2^ = 0.80), with low mean bias (Actual—Predicted) −0.05 sec ± 0.23s, giving 95% limits of agreement (LOA) of −0.50–0.41 s (1.96 × SD). MAE was 0.18 s, and RMSE was 0.19 s. Post hoc recalibration was not required because calibration of predicted versus actual 60 m performances (Actual 60 *m* = 0.023 + 0.992 × Predicted 60 m, *R* ^2^ = 0.80) was close to the identity line. The application of the sample‐maximum correction to a group of U14 athletes is presented in Figure 1C, along with RMSE error bars and model‐based Z‐scores.

The assessment of agreement and prediction error for the sample‐maximum corrections in the long jump was based on 46 observations. Predicted and observed performances showed a moderate association (*R* ^2^ = 0.38), with mean bias (Actual—Predicted) was +0.16 *m* ± 0.44 m, giving 95% LOA were −0.70 m to +1.02 m (1.96 × SD). MAE was 0.37 m, and RMSE was 0.46 m. Post hoc recalibration (Adjusted LJ [m] = 0.939 + 0.829 × Predicted LJ [m]) reduced mean bias 0.00 *m* ± 0.43 m, narrowed 95% LOA ± 0.85 m, improved MAE to 0.33 m and reduced RMSE to 0.43 m. The application of the sample‐maximum correction to a group of U14 athletes is presented in Figure 2C, along with RMSE error bars and model‐based *Z*‐scores.

A descriptive summary of outcome variables presented as the entire sample and split by maturity groups is presented in Table S1 (Supporting Methods).

## 4 Discussion

This study examined the associations of maturity status and chronological age with competition and physical testing performances in male youth track and field athletes, evaluated the effectiveness of mean corrections in reducing maturity‐performance relationships, and assessed the agreement and prediction error in sample‐maximum corrections to a more advanced maturity status. The main findings were that maturity status and chronological age demonstrated outcome‐specific associations with performance across competition and physical test outcomes. In the competition outcomes, maturity status was strongly associated with 60 m performance, whereas maturity status and chronological age were associated with LJ performance. Mean corrections effectively reduced maturity‐related differences in 60 m and maturity‐ and age‐related differences in LJ performance when the correction equation reflected LMM findings. In contrast, the examination of the agreement and error of sample‐maximum corrections differed between the events, with stronger agreement and lower error for 60 m than LJ, highlighting the need for caution when applying this approach.

The first research question examined the independent association of maturity status and chronological age with a range of competition and physical testing outcomes. The LMMs demonstrated that maturity status and chronological age have meaningful outcome‐specific associations to competition (60 m, LJ) and physical testing (CMJ, 50 m, ILP, 10‐5 RJT) performances. These findings are consistent with previous research that identified differences between the least and most mature groups in speed, strength, and power metrics in male youth track and field athletes (Baker et al. 2024) as well as in other sports and youth populations (Meyers et al. 2015; Baker et al. 2025). Conditional *R* ^2^ values indicated that the full models captured a substantial proportion of the variance across all outcomes. However, the lower marginal than conditional *R* ^2^ values indicate that additional variance was explained by between‐athlete random effects beyond the fixed effects of maturity status and chronological age. Partial *R* ^2^ further demonstrated that the unique contributions of the fixed effects varied across outcomes. Practically, these findings indicate that corrective adjustment procedures should be outcome‐specific and guided by the observed contribution of maturity status and chronological age to each performance measure.

In the competition outcomes, partial *R* ^2^ values indicated that that maturity status made a stronger unique contribution to 60 m than LJ, whereas LJ performance was associated with maturity status and chronological age. This may reflect differences in the technical and coordination demands of each event. Straight‐line sprinting may have fewer technical demands than LJ, which requires the athlete to combine approach speed, technical skill, and coordination, particularly during the take‐off phase where horizontal velocity must be effectively converted into vertical velocity. The comparatively weaker findings for LJ may suggest that performance in this event is influenced by factors beyond chronological age and maturity status, including technical proficiency, coordination, strength‐power characteristics, and accumulated event‐specific experience.

In the physical testing outcomes, the statistical significance of the fixed effects (Table 1) and partial *R* ^2^ values (Table 2) showed that the association of maturity status and chronological age differed across variables. Both fixed‐effects were significant predictors of CMJ and 50 m sprint outcomes although partial *R* ^2^ indicated that the stronger predictor varied by outcome. Chronological age made a larger unique contribution to CMJ JH and RP. Across the 10–50 m split times, maturity status and age made similar unique contributions, suggesting that sprint performance under testing conditions may reflect both maturity‐ and age‐related development or training exposure. In contrast, only chronological age was significant for ILP peak force and the 10‐5 RJT outcomes, with partial *R* ^2^ indicating maturity status making comparatively smaller unique contributions.

This may reflect growth‐, maturity‐ and age‐related changes in the muscle (Radnor et al. 2022; Croix et al. 2002; Lexell et al. 1992; Kannas et al. 2010; Gollnick and Matoba 1984; Oertel 1988; Binzoni et al. 2004) and tendon properties (Kubo, Teshima, Hirose, et al. 2014; Kubo, Teshima, Ikebukuro, et al. 2014, 2001) that result in higher peak force and improved stretch‐shortening cycle function in older and more mature athletes (Radnor et al. 2017, 2022; Baker et al. 2021, 2024; Croix et al. 2002). Another plausible explanation, given the athletes' engagement in a rounded development programme, is that chronological age acts as a proxy for training age. In this context, the significant age effects for ILP and 10‐5 RJT may reflect higher scores driven by accumulated training rather than maturation per se. Practically, this highlights the importance of identifying whether maturity status, chronological age or both contribute to an outcome before applying corrective adjustment procedures.

The second research question evaluated the effectiveness of the mean correction approach when applied to competition performances. For 60 m, the mean correction was highly effective, reducing the strong negative correlation between %PAH and 60 m time (*s*) to near trivial as shown in Figure 1A and 1B. For the LJ, the strong positive correlation between %PAH and long jump distance (*m*) was effectively reduced when both maturity status and chronological age were factored into the mean correction (Figure 2A and 2B). These findings show that corrective adjustment procedures can substantially reduce maturity and age‐related performance differences, but the correction applied must reflect the main determinants of performance identified in the LMMs.

The mean corrected performances may offer coaches and practitioners an alternative perspective on youth male competition results after accounting for differences in maturity status and chronological age. For example, corrected results may be used to re‐rank or visualize athletes' results after accounting for maturity status, and where appropriate chronological age, potentially reducing the risk of under‐ or over‐estimating the current performance in less mature and more mature athletes, respectively. However, corrected performances should be viewed as estimates and interpreted cautiously, and considered with other relevant objective (e.g., longitudinal tracking to known benchmarks, physical testing data, training history) and subjective information (e.g., coach observations of technical ability).

The third research question assessed the agreement and prediction error of the sample‐maximum correction approach. Competition performances were corrected to a more advanced maturity status at a subsequent time point and compared with the corresponding observed performances. For the 60 m, the corrected performances showed a strong association with observed performances, and calibration was close to the identity line; therefore, post hoc recalibration was not required. However, the typical prediction error was 0.19 seconds (RMSE), highlighting that corrected times should still be interpreted cautiously and viewed only as an estimate of how an athlete may perform at a more advanced maturity status. For LJ, the association between actual and predicted distances was only moderate, post hoc recalibration was required, and prediction errors were larger (RMSE = 0.43 m). This suggests that forward prediction for long jump performance is more challenging, likely due to the greater technical and coordination demands of the event. When sample‐maximum corrections are presented as applied estimates (Figures 1C and 2C), RMSE should therefore be included to clarify the uncertainty associated with the corrected performance clear.

For the physical test outcomes where agreement and prediction error were not assessed in the present study, the reported SEE values (Table 1) provide an initial indication of the uncertainty should the LMM coefficients be used to derive corrected performances. The distinction between the fixed‐effect SEE and conditional from the LMMs also has practical relevance. Conditional SEE was lower for all outcome variables, reflecting the reduction in unexplained error when repeated measures within athletes were modelled through individual‐level random effects. For example, for CMJ jump height, the conditional SEE (0.02 m) was 50% lower than the fixed effects SEE (0.04 m), while for CMJ relative power, conditional SEE was 48.1% lower than fixed‐effects SEE (2.74 vs. 5.28 W·kg^−1^). Practically, this means that model‐derived corrections may be interpreted with greater precision when repeated measures are available. RMSE from the agreement analysis provides a more direct estimate of prediction error, however, where such an assessment is not available fixed‐effects and conditional SEE provide a means of communicating the uncertainty associated with corrected performances. Presenting corrected performances, alongside an appropriate measure of error is important to help coaches and decision makers appreciate the uncertainty associated with the presented corrections.

When the sample‐maximum correction was applied to the U14 competition data, contrasting patterns emerged for the 60 m and LJ. In the 60 m, all athletes except the athlete representing the sample‐maximum maturity status were predicted to run faster, and the magnitude of the correction exceeded the RMSE with no overlap between raw and corrected times (Figure 1C). This suggests that, for 60 m sprinting, sample‐maximum corrections may provide useful estimates of how 60 m performance could change at a more advanced maturity status, although the uncertainty associated with the corrected performance means they should not be used as the sole basis for any re‐ranking or selection decisions. In the LJ, the larger RMSE resulted in an overlap between the raw and corrected performances for all athletes (Figure 2C). Consequently, the sample‐maximum corrections for LJ should not be used for applied decision‐making and are better viewed as illustrative estimates only.

An applied use of the LMM‐derived coefficients is the utilization of model‐based *Z*‐scores allowing individual performances (Figures 1C and 2C data tables) to be evaluated relative to the expected performance for their current maturity status and chronological age. This avoids the statistical limitations of applying categorizing continuous data (Royston et al. 2006) as well as the uncertainty observed when correcting performances using the sample‐maximum correction. In the LJ examples (Figure 2C), *Z*‐scores of +2 SD identified athletes 3 and 7, who subsequently produced clearly superior performances to the rest of the group in later seasons, with personal bests of 6.96 and 7.00 m at regional and continental under‐18 level. Conversely, very extreme *Z*‐scores (> ± 3 SD) may indicate exceptional talent or potential error in the maturity estimate or measurement. For one 60 m athlete (Figure 1C), an initial *Z*‐score of 3.96 was reduced to 3.06 after recomputing %PAH (91% > 93%) using the known error of ± 2.6 cm in the maturity prediction equation (Lolli et al. 2021), while still classifying them as a high‐performers relative to their maturity status and age. Practically, model‐based *Z*‐scores may help identify athletes performing above or below model expectations for their current developmental status and may also flag potential maturity estimate issues when values fall beyond ± 3 SD and warrant further interrogation of the underlying data.

Several limitations should be considered when interpreting the findings. First, uncertainty from both the maturity assessment and the LMMs is passed into the corrected outcomes and model‐based *Z*‐scores. Each LMM carries residual error and coefficient uncertainty, and the limited number of‐observations prevented reliable evaluation of more complex random‐effects structures, supporting the use of random‐intercept models. Maturity status was estimated using %PAH, which depends on predicted adult height that is subject to individual‐level error. The use of BoneXpert‐derived skeletal age and radiograph‐based predicted adult height represents a methodological strength compared with commonly used maturity‐estimation methods based solely on somatic measurements (Mirwald et al. 2002; Khamis and Roche 1994). Nevertheless, even this approach is reported to have an individual‐level error of ± 2.6 cm (Lolli et al. 2022), which directly affects %PAH and any corrections or Z‐scores derived from it. When extreme values are observed, the underlying data should be interrogated. In practice, %PAH estimates should be accompanied by serial height and body mass measurements, enabling growth velocities to be calculated, which can improve confidence in the maturity estimate. Second, agreement and prediction error of the sample‐maximum corrections were assessed using a moderate sample size with limited repeated measures per athlete. It was also beyond the scope of the present study to conduct an equivalent assessment for all the variables presented here. The 50 m sprint models should also be interpreted cautiously because observations were skewed towards more mature athletes, making their applicability to pre‐ and approaching‐PHV athletes less certain. Third, training age was not quantified, limiting the ability to disentangle training‐ and maturity‐related effects, particularly in more technical outcomes such as LJ. However, because athletes were recruited from a single academy with standardised programing and similar weekly exposure, variability in training stimulus was likely reduced. Future research should externally validate these methods in independent male youth track and field cohorts and examine whether outcome‐specific correction equations remain stable across different events and competitive levels. Given the limited research examining how chronological age, growth and maturation relate to performance in youth female track and field athletes, future studies should also develop and evaluate sex‐specific models in this population.

## 5 Conclusion

This study demonstrated that maturity status and chronological age have outcome‐specific associations with competition and physical testing outcomes in male youth track‐and‐field athletes. Mean corrections substantially reduced maturity‐performance relationships in the competition outcomes when the correction reflected the corresponding associations with maturity status and chronological age identified by the LMMs. In contrast, the agreement and prediction error associated with sample‐maximum corrections differed by event, with stronger agreement and lower error for the 60 m than the LJ, highlighting greater uncertainty in the application of this correction approach. Overall, these findings support the use of outcome‐specific corrective adjustment procedures to enhance the interpretation of male youth track and field performance when maturity‐ and age‐related differences exist. However, corrected performances should be interpreted as estimates and presented alongside an appropriate measure of error to communicate the uncertainty associated with their use in applied decision‐making processes.

## Funding

The authors have nothing to report.

## Ethics Statement

Ethical approval was obtained (E20140000012) from Aspire Zone Foundation (AZF) Institutional Review Board and Research Committee as part of a larger study on growth and maturation in youth athletes.

## Consent

Parental consent and participant assent were obtained prior to commencement.

## Conflicts of Interest

The authors declare no conflicts of interest.

## Supporting information


**Table S1:** Descriptive summary (mean ± SD [*N*]) of outcome variables by whole sample and maturity groups.

## Data Availability

Data supporting this study are not available for sharing. Access is restricted by a data‐sharing agreement with Aspire Zone Foundation, and no additional data can be provided beyond what is reported in the manuscript.
