# Supplementary material for: Enhancing the Interpretation of Competition Performance and Physical Characteristics in Male Youth Track and Field Athletes
Source: Eur J Sport Sci. 2026 Sep 30;26(10):e70259. doi: 10.1002/ejsc.70259 (PMC13627884; doi:10.1002/ejsc.70259)
Supplement: Supplementary file 1 [file EJSC-26-e70259-s001.docx]

**Supplementary Table 1 – Descriptive summary (mean ± SD [N]) of outcome variables by whole sample and maturity groups**

| **Outcome** | **Whole Sample** | **Pre-PHV** | **Approaching-PHV** | **Circa-PHV** | **Post-PHV** |
| --- | --- | --- | --- | --- | --- |
| 60 m Time (s) | 8.56 ± 0.69 [99] | 10.02 ± 0.22 [4] | 9.24 ± 0.52 [21] | 8.55 ± 0.49 [36] | 8.05 ± 0.35 [38] |
| Long Jump (m) | 4.38 ± 0.60 [138] | 3.86 ± 0.21 [4] | 3.95 ± 0.40 [34] | 4.25 ± 0.52 [48] | 4.81 ± 0.49 [52] |
| CMJ Height (m) | 0.33 ± 0.06 [191] | 0.25 ± 0.04 [7] | 0.28 ± 0.04 [42] | 0.32 ± 0.05 [61] | 0.37 ± 0.05 [81] |
| CMJ Relative Power (W·kg) | 50.71 ± 7.07 [191] | 40.90 ± 3.12 [7] | 45.31 ± 5.90 [42] | 49.53 ± 5.30 [61] | 55.02 ± 5.94 [81] |
| Max Velocity (m/s) | 8.58 ± 0.65 [58] |  | 7.91 ± 0.44 [5] | 8.14 ± 0.62 [18] | 8.87 ± 0.50 [35] |
| 10 m Time (s) | 1.78 ± 0.08 [58] |  | 1.88 ± 0.06 [5] | 1.82 ± 0.08 [18] | 1.74 ± 0.06 [35] |
| 20 m Time (s) | 3.09 ± 0.16 [58] |  | 3.29 ± 0.10 [5] | 3.19 ± 0.15 [18] | 3.02 ± 0.11 [35] |
| 30 m Time (s) | 4.34 ± 0.24 [58] |  | 4.64 ± 0.16 [5] | 4.50 ± 0.23 [18] | 4.23 ± 0.17 [35] |
| 40 m Time (s) | 5.58 ± 0.34 [58] |  | 5.98 ± 0.22 [5] | 5.81 ± 0.34 [18] | 5.41 ± 0.24 [35] |
| 50 m Time (s) | 6.83 ± 0.46 [58] |  | 7.36 ± 0.28 [5] | 7.16 ± 0.46 [18] | 6.61 ± 0.32 [35] |
| ILP Peak Force (N) | 2513.90 ± 921.43 [111] |  | 1815.00 ± 620.68 [24] | 2241.63 ± 674.11 [38] | 3025.60 ± 906.15 [49] |
| 10-5 RJT RSI (FT/CT) | 2.92 ± 0.50 [202] | 2.81 ± 0.57 [6] | 2.69 ± 0.37 [39] | 2.83 ± 0.45 [63] | 3.08 ± 0.52 [94] |
| 10-5 RJT Flight Time (ms) | 487.94 ± 50.29 [202] | 449.50 ± 43.71 [6] | 449.62 ± 40.98 [39] | 472.69 ± 40.83 [63] | 515.08 ± 44.10 [94] |
| 10-5 RJT Jump Height (cm) | 29.52 ± 6.01 [202] | 24.99 ± 4.61 [6] | 25.00 ± 4.61 [39] | 27.61 ± 4.69 [63] | 32.78 ± 5.51 [94] |

*Table 2. Abbreviations: PHV = peak height velocity; CMJ = countermovement jump; RJT – repeated jump test; ILP = isometric leg press; FT = flight time; CT = contact time; RSI = reactive strength index*
